# Cross-education via contralateral-limb balance training improves gait speed and sagittal-plane knee motion after anterior cruciate ligament reconstruction: a randomized controlled trial

**DOI:** 10.1186/s13102-026-01878-4

**Published:** 2026-07-11

**Authors:** Chao Liu, ZiHao Sun, Heng Liu, BaoHua Liu, HaiBo Guo

**Affiliations:** 1 Department of Sports Training, Tianjin University of Sport, Tianjin, China; 2 Department of Rehabilitation, Binzhou Medical University Hospital, Binzhou, 256600 China; 3 Department of Special Education and Rehabilitation, Binzhou Medical University, Yantai City, Shandong Province China; 4 Department of Social Sports, Tianjin University of Sport, Tianjin, China

**Keywords:** Anterior cruciate ligament reconstruction, Cross-education, Joint kinematics, Angular velocity, Aangular acceleration, Gait speed

## Abstract

**Objective:**

This study examined whether contralateral-limb balance training, grounded in cross-education theory, enhances gait speed and reconstructed-knee kinematics during early postoperative recovery from anterior cruciate ligament reconstruction (ACLR).

**Methods:**

A single-blind randomized controlled trial enrolled 40 patients 5–6 weeks post-ACLR, randomized 1:1 to an experimental group (*n* = 20; contralateral-limb balance training plus conventional rehabilitation) or a control group (*n* = 20; conventional rehabilitation alone), for 6 weeks (three sessions per week). Lower-limb kinematics during walking were captured using a Vicon three-dimensional motion-capture system, quantifying gait speed and knee-joint range of motion (ROM), peak angular velocity, and peak angular acceleration in the sagittal, coronal, and transverse planes. Data were analyzed using generalized estimating equations (GEE), with analysis of covariance (ANCOVA) examining whether gait-speed changes confounded the kinematic outcomes.

**Results:**

The groups showed no baseline differences (all *P* > 0.05). Gait speed increased in both (both *P* < 0.001), with a significant time × group interaction favoring the experimental group (χ² = 11.98, *P* < 0.001; Hodges–Lehmann estimate 0.08 m/s, 95% CI 0.05–0.11). Sagittal-plane knee ROM likewise improved more in the experimental group (interaction χ² = 9.79, *P* = 0.002; mean difference 8.97°, 95% CI 5.40–12.54°; d = 1.61). No significant interactions emerged for coronal or transverse ROM, or for peak angular velocity or acceleration in any plane (all *P* > 0.05). ANCOVA showed that the sagittal-ROM advantage persisted after adjustment for gait-speed change (*P* = 0.002), indicating an independent benefit, whereas an apparent sagittal peak-angular-velocity advantage disappeared after adjustment (*P* = 0.165). Against available cross-population minimal clinically important difference (MCID) values (no ACLR-specific standards exist), the gait-speed difference fell below the multi-pathology range (0.10–0.20 m/s), whereas the sagittal-ROM difference exceeded the chronic-stroke reference (8.48°), although its 95% CI lower bound did not.

**Conclusion:**

Contralateral-limb balance training, as an adjunct to conventional rehabilitation, yielded greater improvements in gait speed and sagittal-plane knee ROM following ACLR, with the ROM benefit being independent of concurrent gait-speed gains. Kinematic gains were confined to the sagittal plane. Because the between-group differences did not consistently exceed existing MCID thresholds, these findings are statistically robust but of questionable clinical importance, necessitating larger studies and ACLR-specific MCID benchmarks.

**Trial registration:**

Chinese Clinical Trial Registry, ChiCTR2400087325. Registered on 25 July 2024.

## Introduction

The anterior cruciate ligament (ACL) originates from the medial femoral condyle and inserts anterior to the intercondylar eminence of the tibia, where it stabilizes the knee across all three planes of motion alongside the posterior cruciate ligament [1]. Beyond this structural role, the ACL contains a rich mechanoreceptor network, Pacinian corpuscles, Ruffini endings, and others that supply joint proprioception and kinesthetic feedback essential for neuromuscular control [2, 3]. Injury thus damages both mechanical stability and proprioceptive signaling, leading to muscle weakness, impaired motor control, and characteristic gait dysfunction: reduced stance-phase symmetry, shortened stride length, and diminished walking speed that compromises functional independence and quality of life [4–8].

Anterior cruciate ligament reconstruction (ACLR) restores knee anatomy and structural integrity [9]; however, the procedure does not fully or rapidly restore the proprioceptive receptor network and its central integration [2]. Neurophysiological studies show that recovery after ACLR involves a neural reorganization marked by heightened spinal-reflex excitability coupled with reduced corticospinal excitability [10, 11], patterns associated with impaired voluntary quadriceps activation, slower gait, and persistent asymmetry [5, 10, 12]. This neuromuscular lag underlies a critical gap: structural repair proceeds faster than functional recovery, suggesting that targeted neural re-adaptation during early rehabilitation could accelerate kinematic restoration in the reconstructed knee [13]. 

Cross-education (CE) theory addresses this gap [14]. Cross-education describes neural adaptation wherein unilateral training of one limb produces strength or motor-skill gains in the contralateral untrained limb through mechanisms including cortical reorganization, enhanced interhemispheric communication, and bilateral central motor drive [15]. Evidence for CE varies by training type. Resistance training shows consistent support: randomized trials and meta-analytic evidence indicate that training the unaffected limb during early weight-bearing restriction attenuates the postoperative loss of quadriceps strength on the surgical side and improves neuromuscular performance, with a moderate overall effect favoring cross-education [16, 17]. Liu et al. [18] used surface electromyography to confirm that contralateral strength training normalized neuromuscular activation patterns on the affected side. Balance training, however, remains understudied [19]: Karimijashni et al. [20] delivered 8 weeks of neuromuscular training to the non-surgical knee in 30 post-ACLR patients and found improvements in both dynamic and static balance on the reconstructed side, whereas Sabir and Eshan reported no significant between-group differences in pain, balance, or knee-function scores in 58 patients [21]. This disagreement indicates that contralateral balance training’s effects on gait kinematics and speed need verification through adequately powered randomized trials [22].

The present study enrolled patients 5–6 weeks post-ACLR and paired contralateral-limb balance training with conventional rehabilitation. Using three-dimensional motion capture, we measured gait speed and knee-joint ROM, peak angular velocity, and peak angular acceleration in the sagittal, coronal, and transverse planes. We hypothesized that the experimental group would show greater improvements than controls, providing empirical support for applying CE theory to post-surgical lower-limb rehabilitation. We did not incorporate neurophysiological tools, including electroencephalography, transcranial magnetic stimulation, surface electromyography, or neuroimaging, so our findings reflect kinematic-level changes only and cannot directly verify central nervous system mechanisms.

## Methods

### Trial design

We conducted a single-blind randomized controlled trial designed and reported according to CONSORT 2010 guidelines. Patients were recruited between July 2024 and March 2026, with written informed consent obtained before enrollment. An independent statistician generated the randomization sequence using computer-based random number generation. Allocation concealment was maintained using sequentially numbered, opaque, sealed envelopes held by a designated third-party administrator uninvolved in recruitment or treatment delivery; envelopes were opened on-site immediately after baseline assessment. Patients were randomized 1:1 to the experimental group, which received contralateral-limb balance training plus conventional rehabilitation, or the control group, which received conventional rehabilitation alone. Both groups completed a six-week intervention of three weekly sessions (18 total).

Treating physiotherapists could not be blinded to intervention allocation but were excluded from all outcome assessments. Two independent, blinded assessors conducted baseline and post-intervention evaluations in a location separate from treatment areas, without access to allocation records and with explicit instructions not to discuss group assignment with participants; patients received reciprocal instructions. An independent statistician performed all analyses under blinded conditions, with unblinding occurring only after completion. Participants were instructed to maintain their habitual physical activity and diet throughout the trial, with adherence monitored via questionnaires and interviews. Training adherence was calculated as the proportion of attended sessions relative to 18 planned sessions. The supervising therapist documented all adverse events in real time and reported them to the ethics committee according to severity classification.

### Sample size

We estimated sample size a priori using G*Power, referencing Karimijashni et al. [20] with a conservative medium effect size (Cohen’s d = 0.50), two-sided α = 0.05, and power = 0.80, yielding a minimum of 15 per group. Accounting for an anticipated 10% dropout rate brought the requirement to 17 per group; we enrolled 20 per group (40 total) to ensure adequate statistical power.

### Participants

The study was conducted at the Sports Rehabilitation Center of the Affiliated Hospital of Binzhou Medical University. Initial screening by the attending physician assessed postoperative timing and functional status; eligible candidates underwent detailed evaluation by the research team. Of 60 post-ACLR patients screened, two declined enrollment, eight were ineligible (four with serious postoperative complications, two with contralateral lower-limb dysfunction, two with severe cardiopulmonary disease), five had prior ACL injury, and five were excluded for anticipated poor adherence. The remaining 40 were enrolled and provided written informed consent.

*Inclusion criteria*: age 18–45 years, unilateral ACL injury confirmed by imaging, arthroscopic reconstruction with hamstring autograft, independent ambulation without assistive devices within 5–6 weeks postoperatively, and normal contralateral-knee function.

*Exclusion criteria*: prior ACL injury, serious postoperative complications, anticipated poor adherence, severe cardiopulmonary or cognitive disease, and functional impairment of the contralateral lower limb.

All 40 participants were randomized to experimental or control groups (*n* = 20 each), completed the full intervention and follow-up with no dropouts. The Ethics Committee of Binzhou Medical University and its Affiliated Hospital approved the study (Approval No.: 2024-L004; KYLL-239), and the trial was prospectively registered with the Chinese Clinical Trial Registry (ChiCTR2400087325).

### Intervention protocol

The control group received a six-week standardized conventional rehabilitation program. The experimental group received the same program supplemented with contralateral-limb balance training. All sessions were supervised by a qualified physiotherapist and tailored to individual recovery status.

#### Conventional rehabilitation

**Fig. 1:**
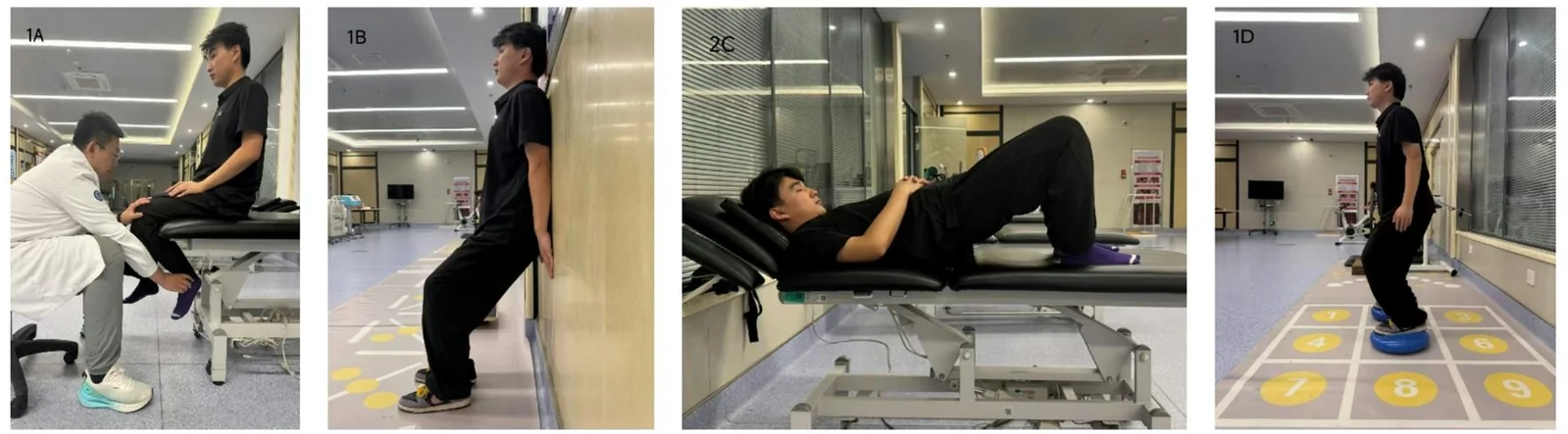
**A**–**D** Conventional rehabilitation exercises

1. *Range-of-motion training*: With the patient seated, the therapist applied moderate posterior pressure at the ankle to guide slow knee flexion until a mild stretch was felt, held for approximately one minute. Once flexion exceeded 90°, prone knee-flexion exercises (10 min per session) progressively restored range while protecting the reconstructed ligament (Fig. 1A).
2. Strength training: Initial sessions comprised supine straight-leg raises with minimal or no resistance to progressively strengthen the quadriceps (≤ 20 min per session). From week three, weight-bearing exercises (wall squats, lunges) were introduced with intensity advancing according to progressive-overload principles (Fig. 1B).
3. Core stabilization training: The foundational exercise was the supine bridge with flexed knees to activate the core and elevate the pelvis until the trunk aligned horizontally (30 repetitions, three sets). As rehabilitation advanced, alternating leg raises and curl-ups enhanced core stability and reduced knee and graft loading (Fig. 1C).
4. Balance and Proprioceptive Training: Initial weight-shifting exercises restored knee proprioception and dynamic postural control, followed by bilateral balance-board training with slight knee flexion to maintain equilibrium (one minute per bout; ≤5 min per session) (Fig. 1D).
5. Stretching and Cool-Down: Static stretching of the quadriceps, hamstrings, and triceps surae concluded each session (20–30 s per muscle, 2–3 repetitions) to relieve tension, improve mobility, and promote local circulation.

#### Contralateral limb balance training protocol

The contralateral-limb balance protocol, adapted from Karimijashni et al. [20], differed from standard balance training by using the non-operative limb as the primary weight-bearing support while fully unloading the operative leg. The program progressed across three phases, systematically increasing postural perturbation and movement complexity.

**Fig. 2:**
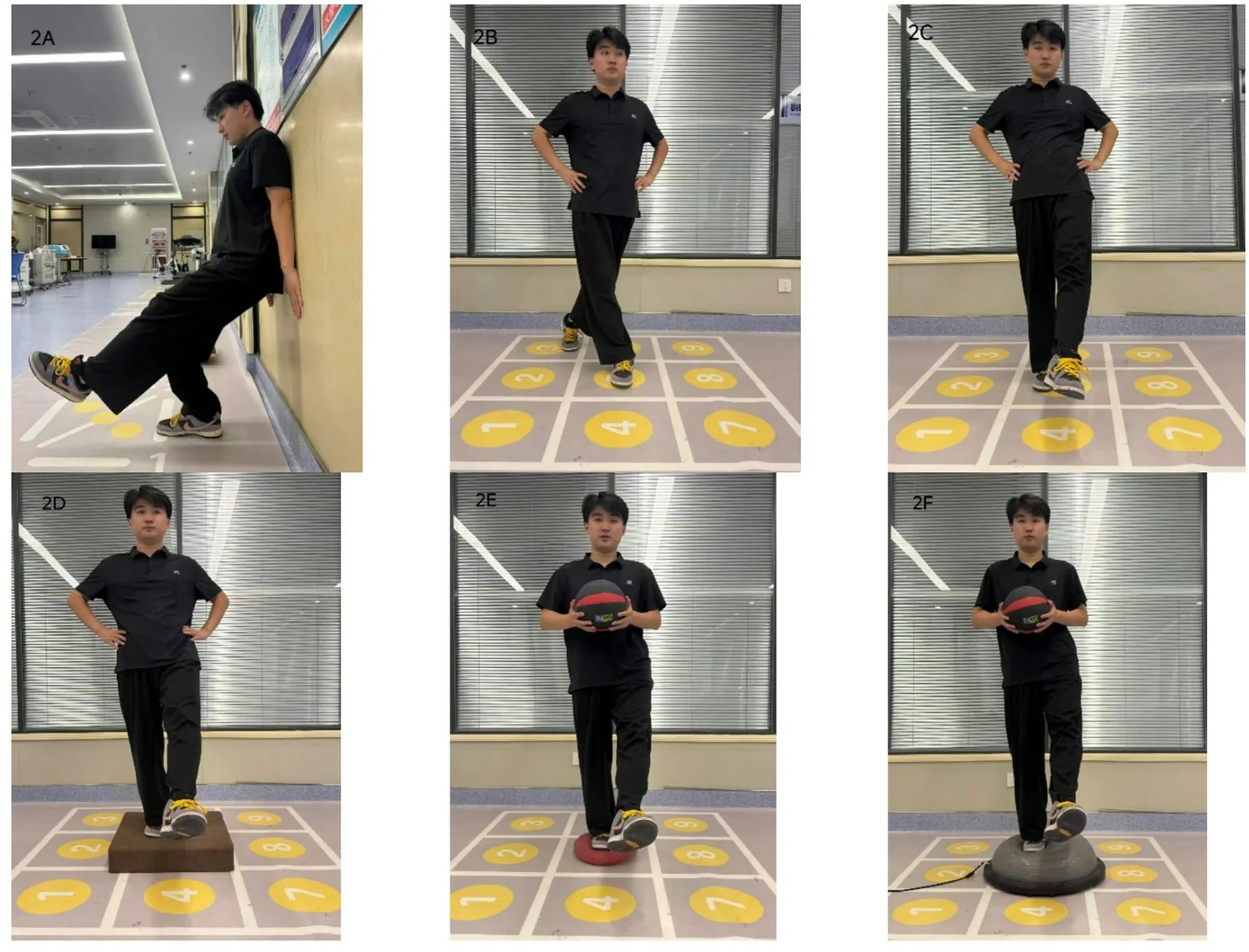
**A**-**F** Three-phase contralateral-limb balance training protocol

- *Phase 1 (Weeks 1–2):* ① Standing with the back against a wall on a stable surface, patients performed single-leg stance on the contralateral limb with the operative leg elevated; eyes-open and eyes-closed balance were each held for 30 s and repeated with slight contralateral-knee flexion (five repetitions per set, 10 sets, one-minute rest intervals) (Fig. 2A). ② Building on this foundation, directional toe-touch exercises followed: with weight supported on the contralateral limb, the operative foot touched down in anterior-left, posterior-left, posterior-right, and anterior-right directions, alternating eyes-open and eyes-closed conditions (five repetitions per set, 10 sets, one-minute rest intervals) (Fig. 2B).
- *Phase 2 (Weeks 3–4):* ① Standing on the contralateral limb with slight knee flexion, patients elevated the operative limb anteriorly and maintained balance with eyes open and closed for 30 s each, then repeated with greater contralateral-knee flexion (five repetitions per set, 10 sets, one-minute rest intervals) (Fig. 2C). ② All Phase 1 exercises were performed on an unstable step platform to increase postural challenge (Fig. 2D).
- *Phase 3 (Weeks 5–6)*: ① Standing on a balance board with the operative limb elevated, patients performed two-handed multidirectional ball tossing and catching for 30 seconds per bout, then repeated with slight contralateral-knee flexion (five repetitions per set, 10 sets, one-minute rest intervals) (Fig. 2E). ② Progressive overload was applied by increasing tossing frequency, augmenting contralateral-knee flexion, or modifying balance-board specifications (Fig. 2F).

The intervention spanned six weeks at three weekly sessions. The treating therapist documented subjective responses and recovery status after each session. All participants completed the full intervention and follow-up without dropout.

### Data acquisition and processing

Lower-limb kinematics during overground walking were captured using a Vicon motion-capture system (eight infrared cameras, 200 Hz; Vicon Motion Systems, Oxford, UK). Participants wore form-fitting laboratory attire to ensure secure marker placement and minimize clothing artifacts. Reflective markers were placed at the anterior and posterior superior iliac spines, lateral mid-thigh, lateral femoral epicondyle, lateral mid-shank, lateral malleolus, first and second metatarsal heads, and superior calcaneus. Anthropometric measurements (height, body mass, leg length, ankle and knee joint widths) were recorded according to Plug-in Gait model requirements. Static calibration and a standing trial were used to initialize the segmental model.

Participants acclimated with three minutes of self-selected walking before completing three trials along an 8-meter walkway at their natural pace without speed constraints [23]. Data from the second trial were analyzed to avoid first-trial acclimatization and last-trial fatigue effects. Gait speed was recorded in real time by the system.

Raw kinematic data were processed in Vicon Nexus using the Woltring quintic spline smoothing algorithm (mean square error = 20), with temporary marker occlusions reconstructed via built-in interpolation. A three-dimensional linked-segment model integrated static calibration with individual anthropometrics. Knee-joint angles were computed in a right-hand coordinate system: flexion–extension (X-axis), varus–valgus (Y-axis), and internal–external rotation (Z-axis). Time-series data for knee angles, angular velocities, and angular accelerations were exported to Python 3.11 for plane-specific range of motion, peak angular velocity, and peak angular acceleration extraction, along with all statistical computations.

### Outcome measures

#### Gait speed

Gait speed (m/s) serves as a direct functional indicator of ambulatory recovery following ACLR. The value from the second valid walking trial was retained for analysis [24].

#### Knee-joint range of motion

Sagittal-plane ROM, reflecting flexion–extension excursion, most directly indicates ambulatory recovery post-ACLR. Coronal-plane ROM characterizes varus–valgus control and lateral stability, while transverse-plane ROM reflects tibiofemoral rotational control and lower-limb kinetic-chain stability [25, 26].

#### Peak knee joint angular velocity

Peak angular velocity (°/s) in each plane indexes the rate characteristics of joint motion and neuromuscular dynamic control. Sagittal values reflect flexion–extension fluidity and muscular co-contraction patterns; coronal values characterize varus–valgus dynamic stability; transverse values indicate tibiofemoral rotational coordination [27].

#### Peak knee joint angular acceleration

Peak angular acceleration (°/s²) indexes rapid neuromuscular recruitment and motor-control precision. Sagittal values reflect rapid force initiation and eccentric deceleration of knee flexor–extensor systems; coronal values characterize instantaneous varus–valgus stabilization; transverse values reflect rapid tibiofemoral rotational responses.

### Statistical analysis

All analyses were performed in Python 3.11. The Shapiro–Wilk test assessed normality; continuous variables were expressed as mean ± standard deviation or median (interquartile range), determined per variable according to normality (a variable was summarized as mean ± SD only when all four group-by-time cells were normally distributed; otherwise as median (IQR)). Baseline comparisons employed the independent-samples *t*-test, Mann–Whitney U test, or chi-square/Fisher’s exact test as appropriate.

Intervention effects were evaluated using generalized estimating equations (GEE) with group as the between-subject factor and time as the within-subject factor, including main effects and the group × time interaction with an independent working correlation matrix. The distributional family was selected by skewness and value range: Gamma with log link for right-skewed knee kinematic variables, and Gaussian with identity link for approximately symmetric gait speed. Wald chi-square statistics and exact *P*-values are reported. When interactions were significant, between-group simple-effects analyses were conducted at each time point (Bonferroni-adjusted α = 0.025). Within-group pre–post differences were assessed using paired t-tests or Wilcoxon signed-rank tests according to normality, with effect sizes expressed as Cohen’s d or the matched-pairs rank-biserial correlation r = (W⁺ − W⁻)/(W⁺ + W⁻), where positive values indicate improvement.

Given that gait speed may influence kinematic changes, sensitivity analyses computed correlations between change in gait speed and change in each kinematic variable using Pearson or Spearman correlation according to normality (Bonferroni-adjusted α ≈ 0.0056). ANCOVA was then fitted with change in each kinematic variable as the outcome, group as the independent variable, and change in gait speed as the covariate; homogeneity of slopes was tested beforehand, with parametric ANCOVA applied when residuals were normal, otherwise rank-transformation-based non-parametric ANCOVA. Confounding was assessed by comparing the group-effect *P*-value before and after adjustment.

Between-group differences in gait speed and sagittal-plane knee ROM change were compared using the independent-samples t-test (mean difference, 95% CI) or Mann–Whitney U test (Hodges–Lehmann estimate, 95% CI) according to normality, then compared against minimal clinically important difference (MCID) thresholds from the literature. All tests were two-tailed with significance set at α = 0.05; exact *P*-values are reported, with very small values denoted *P* < 0.001.

## Results

### Baseline characteristics

Groups did not differ significantly in any characteristic at baseline (*P* > 0.05; Table 1).

**Table 1:** Baseline characteristics by group

Variable	Experimental group	Control group	Statistic	*P*
Age (years)	34.20 ± 4.01	31.60 ± 4.86	*t* = 1.846	0.073
Disease duration (days)	37.90 ± 3.23	36.50 ± 3.36	*t* = 1.343	0.187
Height (cm)	172.30 ± 9.33	167.15 ± 9.11	*t* = 1.766	0.085
Body mass (kg)	65.70 ± 11.43	64.20 ± 14.31	*t* = 0.366	0.716
Sex (male/female)	12/8	11/9	χ² = 0.102	0.749
Involved side (left/right)	12/8	10/10	χ² = 0.404	0.525

### Gait speed

**Fig. 3:**
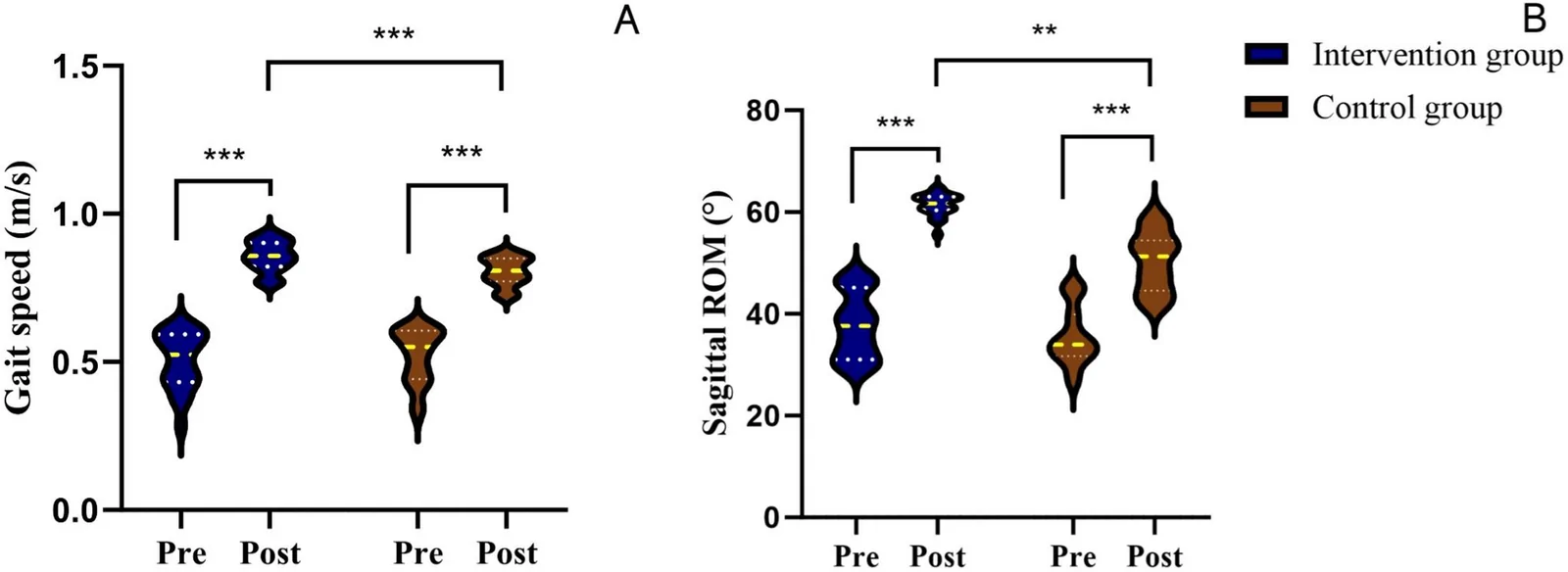
Gait speed (**A**) and sagittal-plane knee ROM (**B**) before and after intervention in the experimental and control groups. Violin plots show the data distribution; the yellow dashed line denotes the median and the white dotted lines the interquartile range. Within-group comparisons (Pre vs. Post) and between-group comparisons at post-intervention are indicated. **P < 0.01, ***P < 0.001

Both groups showed significant within-group improvements in gait speed post-intervention (experimental: *P* < 0.001, *r* = 1.00; control: *P* < 0.001, *r* = 1.00). GEE analysis revealed a significant main effect of time (χ² = 296.90, *P* < 0.001), no main effect of group (χ² = 0.34, *P* = 0.560), and a significant time × group interaction (χ² = 11.98, *P* < 0.001). At baseline, groups were equivalent (*P* = 0.617); post-intervention, the experimental group achieved significantly higher gait speed (*P* = 0.002). The experimental group improved more than controls (Mann–Whitney U = 333.00, *P* < 0.001; Hodges–Lehmann estimate 0.08 m/s, 95% CI 0.05–0.11 m/s), though this difference fell below the MCID range for comfortable walking speed (0.10–0.20 m/s) [28] (Table 2; Fig. 3A).

**Table 2:** Comparison of outcome measures between groups before and after the intervention

Variable	Experimental group			Control group			Time effect	Group effect	Interaction
	Pre	Post	*P* Effect size 95% CI	Pre	Post	*P* Effect size 95% CI	χ² *P*	χ² *P*	χ² *P*
Gait speed (m/s)	0.52 (0.44, 0.59)	0.86 (0.82, 0.90)^***^	*P* < 0.001 *r* = 1.00 (0.32, 0.38)	0.55 (0.45, 0.60)	0.81 (0.78, 0.85)	*P* < 0.001 *r* = 1.00 (0.24, 0.31)	χ² = 296.901 *P* < 0.001	χ² = 0.339 *P* = 0.560	χ² = 11.984 *P* < 0.001
Sagittal ROM (°)	37.61 (31.86, 43.07)	61.68 (60.45, 62.90)^**^	*P* < 0.001 *r* = 1.00 (20.95, 26.98)	33.98 (31.81, 39.30)	51.33 (44.96, 54.25)	*P* < 0.001 *r* = 1.00 (11.61, 17.16)	χ² = 137.593 *P* < 0.001	χ² = 1.253 *P* = 0.263	χ² = 9.79 *P* = 0.002
Coronal ROM (°)	24.56 (14.15, 39.64)	20.21 (16.47, 31.42)	*P* = 0.231 *r* = -0.31 (-11.76, 3.05)	15.21 (10.92, 34.60)	18.93 (16.75, 23.26)	*P* = 0.956 *r* = -0.02 (-11.83, 7.74)	χ² = 0.12 *P* = 0.729	χ² = 0.757 *P* = 0.384	χ² = 0.252 *P* = 0.616
Transverse ROM (°)	17.49 ± 5.43	21.77 ± 7.21	*P* = 0.053 d = 0.46 (-0.06, 8.63)	16.99 ± 6.17	19.05 ± 7.45	*P* = 0.398 d = 0.19 (-2.92, 7.05)	χ² = 0.797 *P* = 0.372	χ² = 0.077 *P* = 0.782	χ² = 0.407 *P* = 0.523
Sagittal peak angular velocity (°/s)	171.82 (147.62, 226.77)	309.89 (289.80, 343.76)	*P* < 0.001 *r* = 1.00 (80.87, 159.44)	156.08 (124.27, 204.65)	252.12 (219.34, 268.06)	*P* < 0.001 *r* = 0.88 (37.18, 98.69)	χ² = 20.524 *P* < 0.001	χ² = 0.443 *P* = 0.506	χ² = 3.765 *P* = 0.052
Coronal peak angular velocity (°/s)	137.57 (78.78, 249.16)	149.15 (107.79, 190.71)	*P* = 0.841 *r* = -0.06 (-56.05, 44.23)	97.37 (55.46, 182.80)	104.69 (74.22, 166.36)	*P* = 0.841 *r*=-0.06 (-57.42, 35.21)	χ² = 0.118 *P* = 0.731	χ² = 1.195 *P* = 0.274	χ² = 0.011 *P* = 0.918
Transverse peak angular velocity (°/s)	111.25 (87.56, 164.20)	184.39 (139.65, 239.56)	*P* = 0.044 *r* = 0.51 (1.46, 103.14)	125.07 (91.49, 171.87)	136.68 (82.51, 170.89)	*P* = 0.927 *r* = 0.03 (-47.98, 44.43)	χ² = 0.001 *P* = 0.976	χ² = 0.411 *P* = 0.521	χ² = 3.292 *P* = 0.070
Sagittal peak angular acceleration (°/s²)	2200.50 (1368.39, 2673.81)	3682.03 (3169.53, 4616.87)	*P* < 0.001 *r* = 0.89 (1031.27, 2612.30)	1699.03 (1462.94, 2520.04)	2403.74 (1848.11, 3657.82)	*P* = 0.015 *r* = 0.61 (205.40, 1718.53)	χ² = 8.851 *P* = 0.003	χ² = 0.113 *P* = 0.737	χ² = 1.926 *P* = 0.165
Coronal peak angular acceleration (°/s²)	1705.21 (1351.63, 3363.14)	2982.73 (1890.07, 4468.89)	*P* = 0.133 *r* = 0.39 (-253.91, 1861.68)	1875.03 (1259.59, 2643.47)	1811.90 (1351.31, 3822.13)	*P* = 0.409 *r* = 0.22 (-423.41, 1301.03)	χ² = 1.648 *P* = 0.199	χ² = 0.13 *P* = 0.718	χ² = 0.371 *P* = 0.542
Transverse peak angular acceleration (°/s²)	2246.59 (1877.80, 2763.15)	3831.35 (3365.34, 4833.42)	*P* = 0.002 *r* = 0.76 (809.27, 2720.76)	2661.81 (1999.65, 3791.06)	2842.48 (1854.00, 3393.70)	*P* = 0.841 *r* = 0.06 (-857.57, 1424.55)	χ² = 0.451 *P* = 0.502	χ² = 0.851 *P* = 0.356	χ² = 3.446 *P* = 0.063

Data are presented as mean ± SD or median (IQR), determined per variable according to normality (a variable is reported as mean ± SD only when all four group-by-time cells were normally distributed; otherwise median (IQR)). Within-group comparisons used the paired t-test (effect size Cohen’s d) or the Wilcoxon signed-rank test (matched-pairs rank-biserial correlation r = (W⁺ − W⁻)/(W⁺ + W⁻), with positive values indicating improvement); the 95% CI refers to the pre–post change. χ² values are Wald statistics from the GEE model

**P < 0.01, ***P < 0.001; asterisks in the Post column denote significant post-intervention between-group differences identified by simple-effects analyses following a significant interaction effect. Simple-effects analyses (Bonferroni-adjusted α = 0.025) showed no between-group differences at baseline; post-intervention, the experimental group was significantly higher for gait speed (t = 3.256, *P* = 0.002) and sagittal ROM (t = 7.910, *P* < 0.001)

### Knee joint range of motion

#### Sagittal plane

Sagittal-plane ROM improved significantly in both groups post-intervention (experimental: *P* < 0.001, *r* = 1.00; control: *P* < 0.001, *r* = 1.00). GEE analysis showed a significant main effect of time (χ² = 137.59, *P* < 0.001), no main effect of group (χ² = 1.25, *P* = 0.263), and a significant time × group interaction (χ² = 9.79, *P* = 0.002). At baseline, groups showed no difference (*P* = 0.323); post-intervention, the experimental group achieved greater sagittal-plane ROM (*P* < 0.001). The experimental group improved substantially more than controls (*t* = 5.09, *P* < 0.001; mean difference 8.97°, 95% CI 5.40–12.54°). The point estimate exceeded the MCID threshold (8.48°) [29], although the lower 95% CI bound (5.40°) did not (Table 2; Fig. 3B).

#### Coronal plane

Coronal-plane ROM did not change significantly in either group (experimental: *P* = 0.231; control: *P* = 0.956). GEE analysis found no significant effects of time (χ² = 0.12, *P* = 0.729), group (χ² = 0.76, *P* = 0.384), or time × group interaction (χ² = 0.25, *P* = 0.616) (Table 2).

#### Transverse plane

Transverse-plane ROM showed no significant change in either group (experimental: *P* = 0.053; control: *P* = 0.398). GEE analysis found no significant effects of time (χ² = 0.80, *P* = 0.372), group (χ² = 0.08, *P* = 0.782), or time × group interaction (χ² = 0.41, *P* = 0.523) (Table 2).

### Peak knee-joint angular velocity

#### Sagittal plane

Both groups showed significant within-group improvements in sagittal peak angular velocity (experimental: *P* < 0.001, *r* = 1.00; control: *P* < 0.001, *r* = 0.88). GEE analysis revealed a significant main effect of time (χ² = 20.52, *P* < 0.001), no main effect of group (χ² = 0.44, *P* = 0.506), and a time × group interaction approaching significance (χ² = 3.77, *P* = 0.052) (Table 2).

#### Coronal plane

Coronal peak angular velocity did not change significantly in either group (*P* = 0.841). GEE analysis found no significant effects of time (χ² = 0.12, *P* = 0.731), group (χ² = 1.20, *P* = 0.274), or time × group interaction (χ² = 0.01, *P* = 0.918) (Table 2).

#### Transverse plane

Transverse peak angular velocity increased significantly in the experimental group (*P* = 0.044, *r* = 0.51) but not in controls (*P* = 0.927). GEE analysis found no significant effects of time (χ² = 0.00, *P* = 0.976), group (χ² = 0.41, *P* = 0.521), or time × group interaction (χ² = 3.29, *P* = 0.070) (Table 2).

### Peak knee joint angular acceleration

#### Sagittal plane

Sagittal peak angular acceleration improved significantly in both groups (experimental: *P* < 0.001, *r* = 0.89; control: *P* = 0.015, *r* = 0.61). GEE analysis showed a significant main effect of time (χ² = 8.85, *P* = 0.003), with no significant main effect of group (χ² = 0.11, *P* = 0.737) or time × group interaction (χ² = 1.93, *P* = 0.165) (Table 2).

#### Coronal plane

Coronal peak angular acceleration did not change significantly in either group (experimental: *P* = 0.133; control: *P* = 0.409). GEE analysis found no significant effects of time (χ² = 1.65, *P* = 0.199), group (χ² = 0.13, *P* = 0.718), or time × group interaction (χ² = 0.37, *P* = 0.542) (Table 2).

#### Transverse plane

Transverse peak angular acceleration increased significantly in the experimental group (*P* = 0.002, *r* = 0.76) but not in controls (*P* = 0.841). GEE analysis found no significant effects of time (χ² = 0.45, *P* = 0.502), group (χ² = 0.85, *P* = 0.356), or time × group interaction (χ² = 3.45, *P* = 0.063) (Table 2).

### Sensitivity analysis and ANCOVA confounding adjustment

Change in gait speed correlated significantly with change in sagittal-plane ROM (Pearson *r* = 0.76, *P* < 0.001), sagittal peak angular velocity (Pearson *r* = 0.50, *P* = 0.001), and transverse peak angular velocity (Pearson *r* = 0.57, *P* < 0.001)—all remaining significant after Bonferroni correction (α ≈ 0.0056). Correlations for the remaining six variables were non-significant after correction. The homogeneity-of-slopes assumption was satisfied for all variables, and ANCOVA adjusting for gait-speed change showed that the group effect on sagittal-plane ROM persisted, indicating the training benefit was independent of concurrent gait-speed gains. Conversely, the group effect on sagittal peak angular velocity, significant unadjusted (*P* = 0.008), became non-significant after adjustment (*P* = 0.165), suggesting this difference was primarily driven by gait-speed improvement rather than a direct training effect. The remaining seven variables showed no significant group effects before or after adjustment (Table 3).

**Table 3:** Sensitivity analysis of the association between change in gait speed and change in each knee kinematic variable, with ANCOVA confounding adjustment

Knee kinematic variable	*r*	*P*	Unadjusted group *P*	Adjusted group *P*
Sagittal ROM (°)	0.763	< 0.001	< 0.001	0.002
Coronal ROM (°)	0.323	0.042	0.589	0.070
Transverse ROM (°)	0.273	0.088	0.486	0.956
Sagittal peak angular velocity (°/s)	0.497	0.001	0.008	0.165
Coronal peak angular velocity (°/s)	0.334	0.035	0.957	0.264
Transverse peak angular velocity (°/s)	0.569	< 0.001	0.071	0.895
Sagittal peak angular acceleration (°/s²)	0.306	0.055	0.096	0.411
Coronal peak angular acceleration (°/s²)	0.380	0.016	0.459	0.514
Transverse peak angular acceleration (°/s²)	0.369	0.019	0.070	0.405

r is the Pearson or Spearman correlation between the pre–post change in gait speed and the change in each kinematic variable (*n* = 40); after Bonferroni correction (α = 0.05/9), the correlations for sagittal ROM, sagittal peak angular velocity, and transverse peak angular velocity remained significant. The unadjusted group P is from a model with group as the only predictor; the adjusted group *P* is from an ANCOVA with change in gait speed as a covariate

For sagittal ROM the group effect remained significant after adjustment (independent effect), whereas for sagittal peak angular velocity it became non-significant, indicating the between-group difference was driven mainly by the gain in gait speed

## Discussion

This randomized controlled trial demonstrated that early postoperative contralateral-limb balance training produces significantly greater improvements in gait speed and sagittal-plane knee ROM compared to conventional rehabilitation alone. Notably, the sagittal-plane ROM benefit persisted after adjustment for concurrent gait-speed gains, indicating an independent training effect; the sagittal peak angular velocity advantage, by contrast, was abolished by the same adjustment. No between-group differences emerged for coronal or transverse ROM, or for peak angular velocity or acceleration in any plane. These findings suggest that contralateral-limb balance training’s kinematic benefit is confined to sagittal-plane ROM and provide preliminary experimental support for applying cross-education theory to early postoperative lower-limb rehabilitation following ACLR. 

Early recovery after ACLR is characterized by persistent neural dysregulation—reduced corticospinal excitability coupled with heightened spinal reflex excitability—that underpins gait asymmetry and restricted sagittal-plane flexion–extension [10, 11]. This imbalance can persist for years post-reconstruction and resists conventional rehabilitation during the weight-bearing restriction phase [11]. Cross-education theory circumvents direct affected-limb loading by leveraging unilateral training to enhance contralateral motor control through adaptive reorganization of corticospinal pathways and coordinated bilateral motor-network modulation [14, 15, 30]. Our intervention targeted the critical 5–6-week window during which this neuro-mechanical imbalance remained pronounced. The significant between-group sagittal-plane ROM advantage provides behavioral evidence that contralateral-limb training facilitates reconstructed-knee recovery through cross-education mechanisms [22].

The plane-specificity of improvements likely reflects the movement composition of the protocol. The training emphasizing single-leg standing, step support, and balance-ball activities predominantly engaged sagittal-plane flexion–extension control, directing neuromuscular stimulus principally in this plane [31]. Sagittal kinematics rely on coordinated quadriceps and hamstring activation through a relatively focused control circuit, plausibly more responsive to six weeks of training; this matches our observed between-group differences [32, 33]. Coronal and transverse movements, by contrast, demand finely coordinated deep stabilizer activation and multisensory proprioceptive integration through more complex neural circuits—processes typically requiring longer cumulative exposure [34–36]. Absence of between-group differences in these planes within six weeks aligns with this mechanistic account. Critically, ANCOVA demonstrated that the sagittal ROM benefit remained significant after adjusting for gait-speed change, confirming independence; the sagittal peak angular velocity advantage did not survive this adjustment, identifying sagittal ROM as the intervention’s primary responsive outcome. Future protocols should systematically incorporate coronal and transverse training components while preserving the sagittal-plane core to achieve more comprehensive three-dimensional kinematic recovery.

The concurrent improvements in gait speed and sagittal-plane ROM provide functional corroboration at the behavioral level. Gait speed, as a direct functional indicator of walking ability post-ACLR, integrates gains across neuromuscular control, joint kinematics, and stability [5, 37, 38]; the convergent directional improvement across both domains supports the value of contralateral-limb training in early rehabilitation. Clinically, however, caution is warranted: the between-group gait-speed difference fell below the pooled MCID range (0.10–0.20 m/s) [28], and while the sagittal ROM change point estimate exceeded a cross-population MCID reference (8.48°) [29], the lower confidence-interval bound did not. No ACLR-specific MCID is currently available. These gains, therefore, are statistically robust but of unclear clinical significance pending population-specific benchmarking.

Contralateral-limb balance training, wherein the unaffected limb provides primary support while the affected limb remains unloaded, proved feasible and practicable during weight-bearing restriction, was well tolerated with perfect adherence and no dropouts, and offers a structured, standardizable three-stage protocol. Given the verified independence of sagittal-plane ROM improvement from gait-speed change, this modality warrants incorporation as an adjunct to conventional early rehabilitation, tailored to individual needs and phase-specific recovery milestones.

## Limitations

Several limitations merit discussion. The six-week intervention duration prevents assessment of long-term kinematic maintenance; extended follow-up is necessary. Outcomes were restricted to knee kinematics and gait speed, excluding spatiotemporal parameters, joint kinetics, muscle activation, and patient-reported measures, limiting functional comprehensiveness. Neurophysiological measures—electroencephalography, transcranial magnetic stimulation, neuroimaging—were not employed, so findings reflect kinematic-level changes only without direct verification of central nervous system mechanisms; multimodal neurophysiological assessment should be integrated into future work. The experimental group received additional training volume, therapist contact, and balance-stimulus intensity relative to controls, confounding the specific intervention effect; an active control group with matched training volume would isolate dose-independent effects. Finally, the modest sample size may have insufficient power to detect true between-group effects in coronal and transverse planes; replication in larger cohorts is warranted.

## Conclusion

Contralateral-limb balance training as an adjunct to conventional rehabilitation yielded greater improvements in sagittal-plane knee ROM and gait speed following ACLR. ANCOVA confirmed the sagittal-plane ROM benefit was independent of concurrent gait speed gains, with kinematic advantages confined to the sagittal plane. The protocol requires no weight-bearing on the affected limb, making it feasible, straightforward, and well-tolerated, with complete adherence and no dropout, and suitable for the weight-bearing restriction phase of early recovery. Since between-group differences did not consistently exceed existing cross-population MCID thresholds, these gains are statistically robust but of uncertain clinical magnitude. These results provide preliminary experimental evidence for translating cross-education theory into postoperative lower-limb rehabilitation practice. Larger-scale studies with extended follow-up and multimodal neurophysiological assessment are needed to clarify underlying neuroregulatory mechanisms and optimize clinical application.

## Data Availability

Any data will be available at the corresponding author upon request.
